# Squamous cell carcinoma (SCC) of the Pyriform sinus with multiple metachronous brain metastases, a case report

**DOI:** 10.1186/s13014-020-1472-0

**Published:** 2020-01-20

**Authors:** Agostino Cristaudo, Antonio Stefanelli, Stefano Ursino, Durim Delishaj, Davide Baldaccini, Alessandra Gonnelli, Fabiola Paiar

**Affiliations:** 1 Department of Radiotherapy, Azienda Ospedaliero-Universitaria di Ferrara, Via Aldo Moro, 44124 Ferrara, Cona Italy; 2 Department of Radiotherapy, Azienda Ospedaliero-Universitaria Pisana, Pisa, Italy; 3 Department of Radiation Oncology, “A. Manzoni” Hospital, Lecco, Italy

**Keywords:** Head and neck carcinoma, Brain, Metastases, Radiotherapy

## Abstract

**Background:**

Distant Metastases from Head and Neck Squamous cell carcinomas are uncommon (9–11%) and they are usually found in the lung and less frequently in the liver, kidney and adrenals.

Central nervous system (CNS) metastases are extremely rare (2–8%), and they are described mainly in patients who already have extracranial metastases. So there’s scarcity of data about their optimal management .

**Methods and results:**

A patient presented CNS metastases after having been successfully treated with induction chemotherapy and definitive radiotherapy for a pyriform sinus carcinoma. The patient’s work up, treatment and outcome are described.

**Conclusions:**

CNS metastases from Head and Neck carcinomas are exceptionally rare. Nevertheless, clinicians should be alert of neurological symptoms in these patients, in order to set up a timely assessment and treatment. Secondarily, given the rarity of this condition, additional research on this topic is warranted in order to improve therapeutic strategies and outcomes of such patients.

## Main Text

This is the case study of a 52 - year - old Caucasian woman with hypothyroidism, strong smoker (68 pack/years) and with a history of heavy alcohol consumption and anxious-depressive syndrome.

During routine follow up for hypothyroidism an enlarged neck node was found. A Fine Needle Aspiration Cytology (FNAC) was performed with the diagnosis of metastasis from squamous cell carcinoma.

A Computed Tomography (CT) scan identified at least 3 right laterocervical lymphadenopathies and a thickening of the contiguous area of the hypopharynx and of the nasopharynx. A subsequent Fluorodeoxyglucose-Positron Emission Tomography/Computed Tomography (FDG-PET-CT) documented hypercaptation in the laterocervical lymphadenopathies and in the right laryngeal ventricle, showing no hypercaptation in the nasopharynx. An esophagogastroscopy didn’t show any suspicious finding, while an otolaryngology consult documented a neoplastic lesion in the right pyriform synus, which underwent biopsy resulting a Grade 2 squamous cell carcinoma. The nasopharynx was negative.

The proposed clinical stage was cT2 cN2b.

The patient therefore underwent 3 cycles of neoadjuvant chemotherapy (Docetaxel, Cisplatin and 5-Fluorouracil, TPF) and then she was treated with definitive RT.

A dose of 54 Gy in 2 Gy fractions was administered to the laterocervical nodes (Levels from II to V, bilaterally), while the tumor in the right pyriform sinus and the right laterocervical lymphadenopathies received a total dose of 70 Gy in 2 Gy fractions with sequential boost. The treatment was delivered with Volumetric Modulated Arc Therapy (VMAT), in an overall time of 53 days.

Noticeably, the patient continued smoking and drinking alcohol during the treatment and the follow up.

**Fig. 1:**
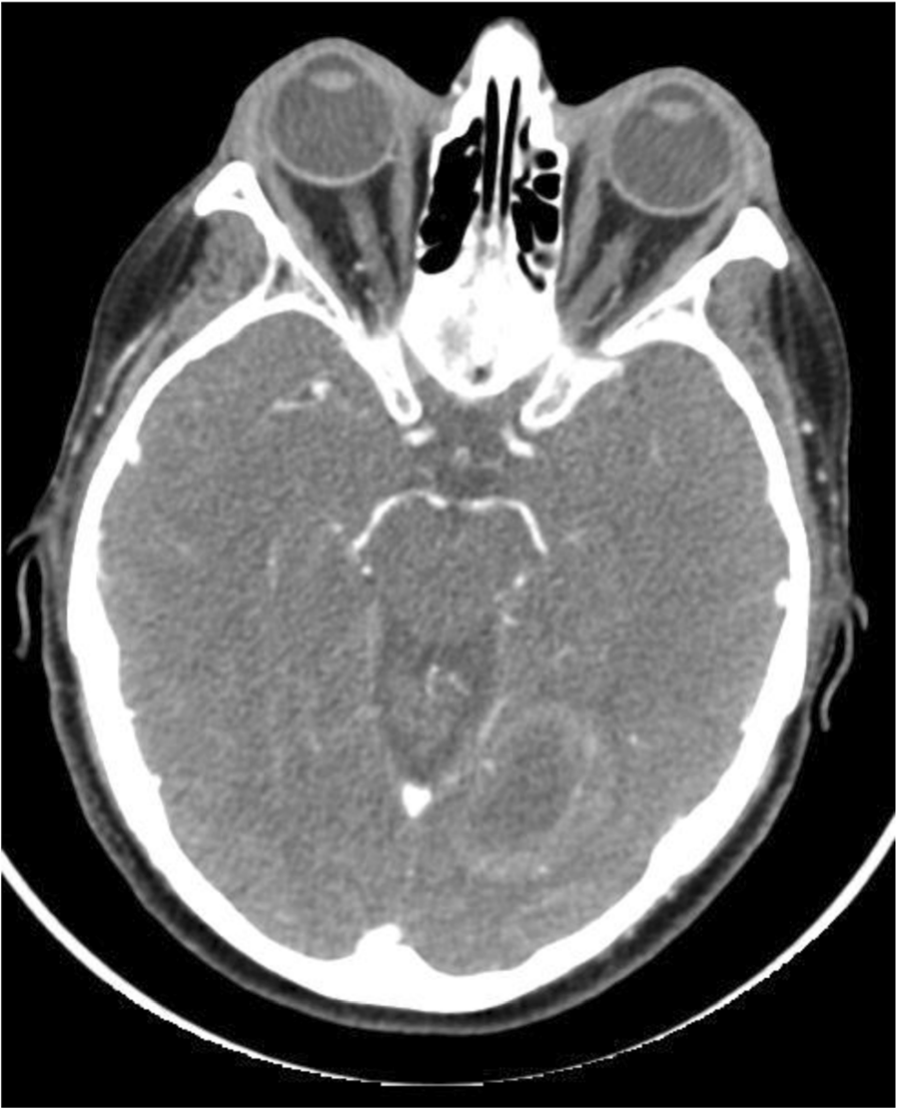
CT Scan of the space occupying lesion found in the left occipital Lobe of the brain

The patient remained completely asymptomatic and NED for 6 months; then a CT scan showed a space occupying lesion in the left occipital lobe of the brain. As the CT scan aimed at assessing the tumor response on the primitive site and the neck, it included only a small part of the brain (Fig. 1).

**Fig. 2:**
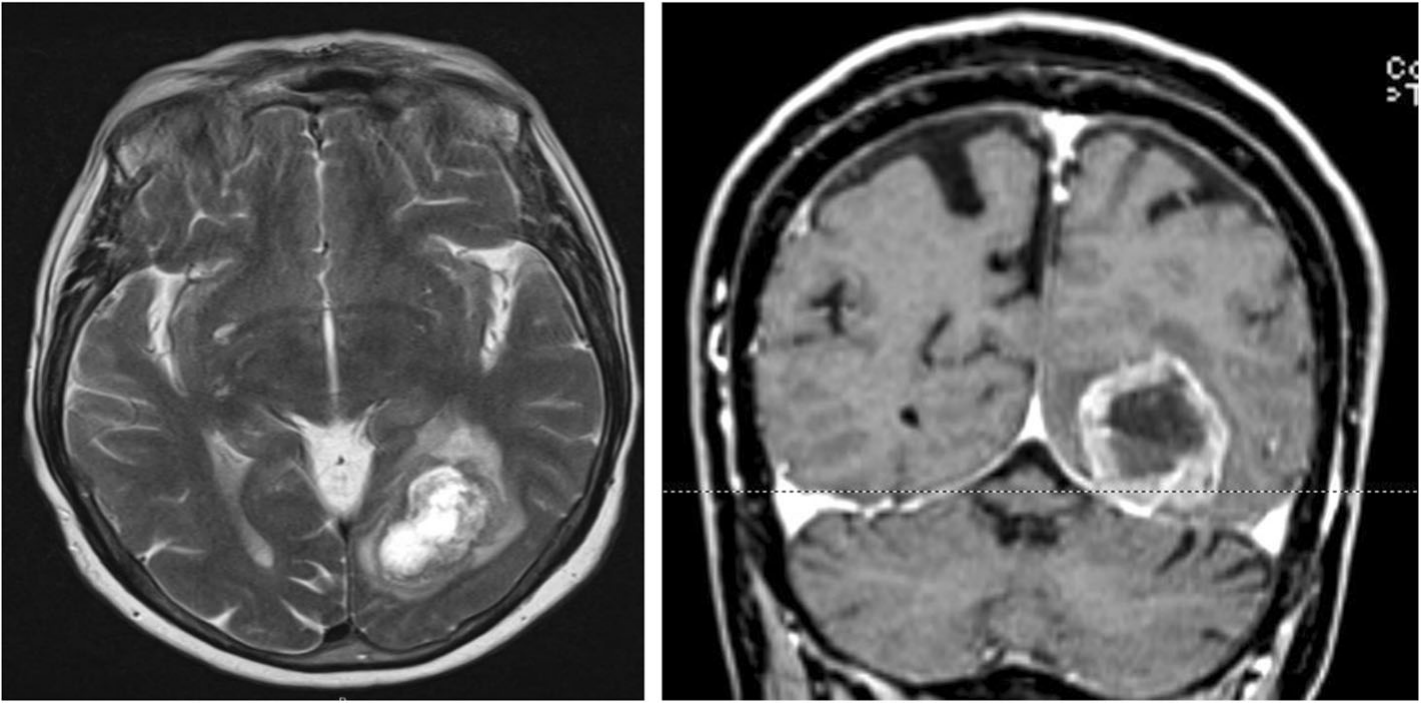
Mr of the same lesion

A subsequent Magnetic Resonance (MR) (Fig. 2) confirmed the lesion but its features suggested a primitive brain malignancy, so it was surgically removed.

Differently from the radiologic features of the lesion, the immunopathologic studies on the surgical specimen proved it was a metastasis from a Squamous Cell Carcinoma, so a workup to detect the primary was undertaken. A PET-CT scan was completely negative, while a gastroscopy, a colonoscopy and a bronchoscopy have been refused by the patient.

**Fig. 3:**
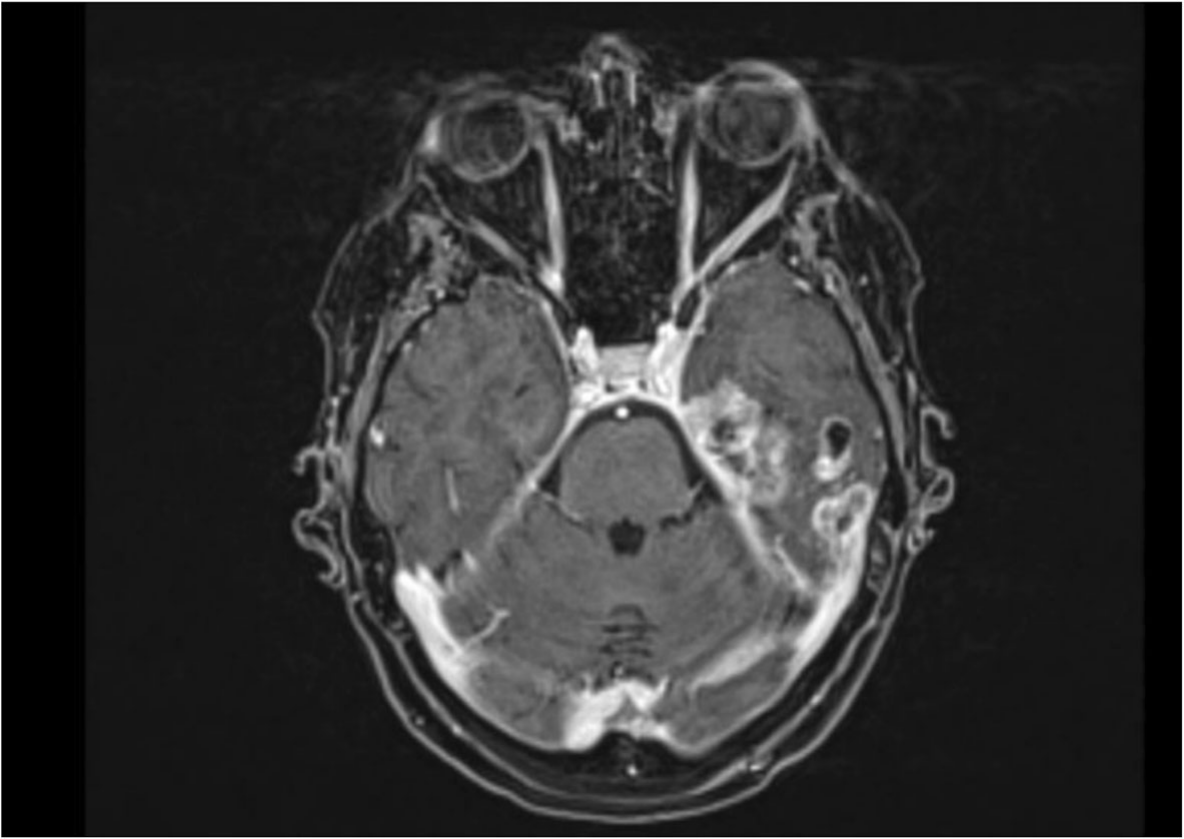
Subsequent Mr showing disease relapse and additional Metastases

4 months later, because of neurologic symptoms, mainly memory loss and disorientation, a new brain MR (Fig. 3) was requested with evidence of disease relapse near the site of excision and other brain metastases, for the most part in the left temporal lobe (in the meantime there have been no relapse at the pyriform sinus and the neck nodes).

A palliative brain radiotherapy was therefore planned (25 Gy in 2,5 Gy fractions on the whole brain with a simultaneous integrated boost to a total dose of 35 Gy in 3,5 Gy fractions on the brain metastases), but because of worsening neurologic symptoms the treatment was suspended and the patient was started on Best Supportive Care till the exitus.

## Discussion and teaching point(s)

Squamous cell carcinomas of Head and Neck (HNSCC) are the 6th most common cancer globally [1].

The percentage of distant metastases is 9–11% [2], and such percentage varies according to the neoplasm’s anatomical district [2], the hypopharynx being one of the sites with the highest probability [3].

The commonest sites of distant metastases are the lungs then the liver [2] and subsequently kidneys and adrenals [4]. Brain is an extremely rare site of metastases from HNSCC [2–6]; a percentage of 2–8% is reported in patients who already have extracranial metastases [2]. (only 1% of brain metastases are from HNSCC origin [5]), consequently there’s shortage of works on their management.

For instance, De Bree et al. [2] have described in their center 13 cases of brain metastastes in a total of 5141 HNSCC patients. 7 of them have been treated with Whole Brain Radiation Therapy (WBRT), 1 with Stereotactic Radiation Therapy (STR), and 5 didn’t receive active therapies. In particular, the Authors reported on a 53 - year - old man with laryngeal carcinoma treated with surgery and adjuvant radiation with a histologically proven metastasis in the left cavernous sinus. Such lesion has been treated with radiosurgery (15 Gy) and the patient remained SD for 5 months.

Djalillan et al. [4] reported on 5 cases of brain metastases from HNSCC. One of them has been treated with surgery only, 3 received exclusive radiation (2 WBRT and one has been treated on cerebellum and skull base) and one underwent surgical metastasectomy and adjuvant WBRT.

Dodelbower et al. [6] have published the case of a woman with tonsil carcinoma with a synchronous brain metastasis, which underwent resection and subsequent brachytherapy (Gliasite balloon catheter to a dose of 45 Gy), while the primary disease was treated with chemoradiation. 2 months after, the patient developed 2 additional brain metastases, treated with Gamma-Knife Radiosurgery.

Even if HNSCC patients with brain metastastes are rare, it should be underlined that, because of more effective therapies, survival is increasing even in this setting, so in the future we could face an increased incidence of such metastases [5].

Risk factors for distant metastases in HNSCC are: T and N staging, ECE [Extra Capsular Extension], positive resection margins, lymphovascular and perineural invasion [2]. Prognosis of HNSCC patients with intracranial metastases is poor, with a mean survival of 4,3 months [2].

As far as therapy, main strategies are surgery and radiotherapy, since most drugs cross the Blood Brain Barrier [5] poorly.

For multiple and diffused brain metastases the commonest therapeutic strategy is WBRT which, even being a palliation, is effective in improving neurological symptoms and it diminishes the possibility of additional intracranial metastases [7].

In the event of oligometastatic disease in the brain, surgical excision should always be considered, as it’s been showed to improve survival [8].

Additionally, the effectiveness of an adjuvant radiation treatment has been documented [8].

Traditionally, the most usual radiation treatment even in this setting has been WBRT. Its effectiveness has been shown in lowering local and brain recurrence rates and the risk of death for neurological causes [9, 10], but it has several side effects, such as alopecia, nausea and asthenia, and above all neurocognitive decline.

The use of SRT or even radiosurgery in the adjuvant setting (on the surgical bed) has been suggested [8, 11], .also with the purpose of lowering such side effects.

A Randomised, Controlled phase 3 Trial has shown that postoperative SRT for brain metastases confers the same oncological outcomes of WBRT, with less neurocognitive toxicity [7].

### Teaching point(S)

1. Clinicians should be particularly alert to neurologic symptoms in patients with HNSCC above all if they have one or more of the aforementioned risk factors for distant metastases; these symptoms should lead to a timely neurological evaluation with proper imaging and a prompt therapy, with the purpose of improving an otherwise poor prognosis.
2. As the cases of brain metastases from HNSCC, albeit extremely rare, are likely to increase, research on this topic should be encouraged with the aim of reaching a deeper knowledge and developing more effective therapies.

## Data Availability

all the Patient’s data and medical images can be found on the database of Pisa Hospital.

## Abbreviations

HNs: Head and Neck
SCC: Squamous Cell Carcinoma
CNS: Central nervous system
CT: Computed Tomography
ECE Extra: Capsular Extension
FDG-PET-CT: Fluorodeoxyglucose-Positron Emission Tomography/Computed Tomography
FNAC: Fine Needle Aspiration Cytology
Gy: Gray
MR: Magnetic Resonance
NED: No Evidence of Disease
RT: Radiotherapy
SCC: Squamous cell carcinoma
STR: Stereotactic Radiation Therapy
TPF: Docetaxel, Cisplatin and 5-Fluorouracil
VMAT: Volumetric Modulated Arc Therapy
WBRT: Whole Brain Radiation Therapy
